# Lactoferrin exhibits PEDV antiviral activity by interfering with spike-heparan sulfate proteoglycans binding and activating mucosal immune response

**DOI:** 10.1186/s13567-025-01456-5

**Published:** 2025-01-31

**Authors:** Peng Liu, Jinjiao Zuo, Hui Lu, Bin Zhang, Caihong Wu

**Affiliations:** 1 College of Veterinary Medicine, Jiangsu Agri-Animal Husbandry Vocational College, Taizhou, 225300 Jiangsu China; 2 Pet Science and Technology College, Jiangsu Agri-Animal Husbandry Vocational College, Taizhou, 225300 Jiangsu China

**Keywords:** Lactoferrin, PEDV, heparan sulfate proteoglycans, mucosal immunity, neonatal piglets

## Abstract

Neonatal piglets infected with Porcine Epidemic Diarrhea Virus (PEDV) experience a mortality rate of up to 90%, resulting in significant economic losses to the swine industry in China. Current strategies using specific antibodies in sow milk to prevent Porcine Epidemic Diarrhea (PED) in these piglets through specific antibodies in sow milk is unsatisfactory. Preliminary studies have shown limited success. Emerging evidence suggests that general immune factors in sow milk provide protection to neonatal piglets, particularly, lactoferrin, play a crucial role in protecting piglets by inhibiting PEDV replication. However, the precise mechanism by which lactoferrin exerts its antiviral effects remains unclear. This study sought to clarify these mechanisms through both in vitro and in vivo approaches, proposing that higher concentrations of lactoferrin lead to greater antiviral activity. It was hypothesized that lactoferrin can impede PEDV by blocking its binding to heparan sulfate proteoglycans (HSPG) on the surface of target cells, and molecular docking experiments was conducted to identify the binding sites between lactoferrin and HSPG. Additionally, the findings indicated that lactoferrin can effectively trigger the maturation of porcine dendritic cells and boosts their antigen-presenting functions, thereby improving intestinal mucosal immunity in neonatal piglets against PEDV. Overall, these findings aid to elucidate the antiviral actions and mechanisms of lactoferrin in sow colostrum, offering new insights for the effective prevention and control of PED in neonatal piglets.

**Supplementary Information:**

The online version contains supplementary material available at 10.1186/s13567-025-01456-5.

## Introduction

Porcine epidemic diarrhea (PED) is an infectious gastrointestinal disease in swine caused by the Porcine epidemic diarrhea virus (PEDV). The disease is characterized by severe watery diarrhea and is notorious for its high morbidity, rapid spread, and devastating mortality rates, with neonatal piglets experiencing mortality rates of up to 90% upon infection with PEDV [1]. Currently, most vaccines aimed at preventing PED are delivered through intramuscular injections. However, these vaccines often fail to provide effective immunity in time due to the immature immune systems of neonatal piglets, leaving them highly vulnerable to PEDV infections [2]. Studies have shown that orally immunizing pregnant sows with PEDV can boost specific antibody levels in their milk, which in turn can significantly lower the mortality rates among neonates [3]. Therefore, vaccinating sows to confer immunity to their offspring is seen as the most viable prevention method. However, the rapid mutation rate of the PEDV S gene presents a challenge, as it causes a delay in vaccine development that struggles to keep up with viral evolution, complicating efforts to manage PED in swine populations [4]. Even though orally immunized sows produce high levels of specific antibodies in their milk, piglets still exhibit symptoms of diarrhea when challenged with heterologous PEDV strains, suggesting that while some cross-protection exists among PEDV subtypes, it is not entirely effective [5].

In addition to specific antibodies, innate immune components in milk, such as cytokines and antiviral proteins, are crucial in fighting off viral infections in the intestine [6]. Sow milk is differentiated into colostrum and mature milk based on the time of secretion. Colostrum, unlike mature milk, is abundant in immune factors, including antibodies, immune cells, cytokines, and antiviral proteins [7, 8]. Lactoferrin (LF) is an iron-binding glycoprotein predominantly present in mammalian colostrum [9]. LF not only inhibits viral invasion but also modulates the host's immune response [10, 11]. Although prior studies have shown impact of LF on PEDV replication [10], the specific mechanisms of its antiviral action against PEDV remain to be fully understood. Therefore, in this study, we first conducted in vitro experiments to explore how LF inhibits PEDV replication by blocking its binding to heparan sulfate proteoglycans (HSPG) on the surface of target cells. Additionally, we investigated how LF can enhances the intestinal mucosal immunity of neonatal piglets to protect them from PEDV infection. In summary, this project has elegantly elucidated the molecular mechanisms by which LF prevents PEDV infection in neonatal piglets. These findings provide a solid theoretical foundation for developing strategies to prevent and control PED in neonatal piglets, offering significant insights into the role of LF in enhancing intestinal mucosal immunity and inhibiting viral replication.

## Materials and methods

### Strain and cell lines

The wild-type PEDV strain and its susceptible Vero E6 cells were kindly provided by Professor Li Zhang from our laboratory. These cells were grown and maintained in DMEM supplemented with 10% fetal bovine serum.

### Experimental animals and grouping

To assess the safety of orally administered LF, acute toxicity tests were performed on mice. For this study, BALB/c mice aged 4 weeks were divided into five groups, each consisting of five mice. Following a 3-day period of acclimatization with standard feeding, the mice were given varying concentrations of LF solutions daily through gavage for a week. The administered concentrations included 0, 100, 200, and 400 mg/kg. Mortality was monitored daily in all groups.

The experimental pigs used in this study were crossbred pigs (Duroc × Landrace × Large White), sourced from the pig farm at the Jiangsu Academy of Agricultural Sciences. Before the experiments began, these pigs tested negative for antibodies against Porcine Epidemic Diarrhea Virus (PEDV), Porcine Reproductive and Respiratory Syndrome Virus (PRRSV), Porcine Respiratory Corona Virus (PRCV), Transmissible Gastroenteritis Virus (TGEV), and Porcine Circovirus Type 2 (PCV2). Twenty-five healthy newborn piglets were randomly selected from the pig farm and were continuously fed commercially available milk powder for 7 days. Subsequently, on the 5th day of age, the piglets were inoculated with PEDV (10^4^ PFU/mL). One hour before the PEDV inoculation, they were orally administered either PBS or LF. The control group consisted of piglets that were orally administered 1 mL of PBS but were not inoculated with PEDV. The positive control group consisted of piglets that were orally administered 1 mL of PBS and were inoculated with PEDV. In the LF1 group, piglets were orally administered 1 mL of commercially available LF (100 mg/kg). In the LF2 group, piglets received 1 mL of LF (200 mg/kg), and in the LF3 group, they received 1 mL of LF (400 mg/kg). All three groups were also inoculated with PEDV. Euthanasia was performed on all piglets 48 h post-infection. Fecal samples were collected from the piglets at 0, 6, 12, 24, 36, and 48 h post-infection, and tissues from the duodenum, jejunum, and ileum were obtained for relevant assays. Additionally, the pathological changes in the intestinal tract were observed from 0 to 48 h after infection and were scored according to the severity of diarrhea. The scoring criteria were as follows: 0 indicated that the stool was hard and shaped, 1 indicated that the feces were soft and shaped, 2 indicated that the feces were semi-solid without shape, a score of 3 indicated watery diarrhea.

### Detection of cytotoxic effects of HSPG and the LF and inhibitors on cells

In this study, the cytotoxic effects of HSPG, LF, and their inhibitors were assessed using the CCK-8 assay kit, following the manufacturer’s protocol.

### Effects of HSPG or LF on PEDV replication

Vero E6 cells were initially incubated with different concentrations of HSPG (10, 100, 250, and 500 µg/mL), LF (100, 250, and 500 µg/mL), and LF inhibitors (1, 25, and 50 µM) at 37 °C for 1 h. The cells were then washed with DMEM to remove any residual compounds. After this, PEDV at a concentration of 10^4^ PFU/mL was added to the cells and incubated first for 1 h at 4 °C and then at 37 °C. The cells were again washed with DMEM to eliminate any unattached virus, and then 1 mL of DMEM culture medium was added to each well. After 24 h, the supernatant and RNA samples were collected for conducting plaque reduction neutralization tests and RT-qPCR analysis. The viral load of PEDV in the cells was quantified using relative quantitative PCR, with GAPDH serving as the reference gene and the PEDV-N gene as the target gene. The primer sequences used are listed in Table 1.

**Table 1:** **The primers used in this study**

Gene	Primer forward/Reverse	Length
PEDV-N-F	AAGGCGCAAAGACTGAACCC	20 bp
PEDV-N-R	TGTTGCCATTACCACGACTCC	21 bp
IL-1β-F	GCCAACGTGCAGTCTATGGAGTG	23 bp
IL-1β-R	GGTGGAGAGCCTTCAGCATGTG	22 bp
IL-6-F	AAATGTCGAGGCCGTGCAGATTAG	24 bp
IL-6-R	GGGTGGTGGCTTTGTCTGGATTC	23 bp
GAPDH-F	TCATCATCTCTGCCCCTTCT	20 bp
GAPDH-R	GTCATGAGTCCCTCCACGAT	20 bp

### Immunofluorescence assay

#### Co-localization of PEDV and HSPG

Cells were seeded onto coverslips and incubated until confluent. After this, the cells were exposed to PEDV for two hours, then washed and blocked with 5% BSA. Subsequent to five thorough PBS washes, the cells were treated overnight at 4 °C with rabbit anti-HSPG (bs-5072R, Bioss, China) and mouse anti-PEDV (PEDV-N, Medgenelabs Biological, USA) primary antibodies. After additional washes with PBS, the cells were incubated for 2 h with Alexa Fluor 594-conjugated goat anti-rabbit IgG (bs-0295G-AF594, Bioss) and FITC-conjugated goat anti-mouse IgG (bs-0296G-FITC, Bioss) secondary antibodies. Following five more washes with PBS, DAPI staining was applied to visualize the nuclei. The co-localization of PEDV and HSPG on the cell surface was then examined using a fluorescence confocal microscope.

#### Co-localization of LF and HSPG

LF was initially incubated with Vero cells at 37 °C for one hour, after which the cells were washed using DMEM. Subsequently, PEDV (10^4^ PFU/mL) was inoculated into the cells and incubated at 4 °C and 37 °C for 1 h each. The cells were then washed and blocked with 5% BSA. Thereafter, the cells were stained with specific antibodies against LF (mouse anti-lactoferrin antibody, bsm-51024M, Bioss) and HSPG, along with fluorescently labeled secondary antibodies. The co-localization of LF and HSPG on the cell surface was confirmed using a fluorescence confocal microscope.

### Screening of interaction sites between LF and HSPG

To further identify the binding sites between lactoferrin and HSPG, this study employed homology modeling to predict their three-dimensional structures, followed by molecular docking (protein–protein) to determine the optimal binding conformation between LF and HSPG. The specific procedures were conducted by an external specialized company. Then, based on the results from the molecular docking, site-specific LF mutants were generated, employing the Mut Express® II Fast Mutagenesis Kit V2 (C214-01, Vazyme, China) as per the manufacturer's instructions. Subsequently, these mutants were incubated with Vero cells in a 37 °C incubator for 1 h, followed by washing with DMEM medium. PEDV (10^4^ PFU/mL) was then administered to the cells, which were incubated sequentially for 1 h at 4 °C and then at 37 °C. Following this incubation, the cells were again washed with DMEM to eliminate any residual virus, and 1 mL of DMEM culture medium was added to each well. After a period of 24 h, both supernatant and RNA samples were harvested to conduct plaque reduction neutralization tests and RT-qPCR analysis.

### Isolation of porcine dendritic cells and T lymphocytes

#### Isolation of porcine dendritic cells

Porcine dendritic cells (DCs) were obtained by differentiating bone marrow-derived monocytes through in vitro induction and culture. The detailed experimental procedures are described below:

Piglets were euthanized, and their femurs were removed and transferred to a sterile laminar flow cabinet. The femurs were first sterilized in 75% ethanol and then in saline solution for 5 min each. Subsequently, they were immersed in 2% antibiotic 1640 medium for 15 min. Using a bone marrow puncture needle, the top of the femur was pierced, and red bone marrow was aspirated using a 50 mL syringe. This bone marrow was then diluted in culture medium, and the cells were gathered by centrifugation at 1500 rpm for 5 min. Red blood cells were removed using a red blood cell lysis buffer, and the remaining cells were washed twice with culture medium. Next, cells were seeded at a density of 2 × 10^6^ cells/mL into each well of a 6-well culture plate. They were cultured in medium enriched with porcine GM-CSF (20 ng/mL) and IL-4 (10 ng/mL), and incubated under standard conditions. The medium was changed every 3 days. After 6 days, all non-adherent and semi-adherent cells were collected for subsequent experiments.

#### Isolation of porcine T lymphocytes

Porcine mesenteric lymph nodes were collected and processed to extract lymphocytes as detailed below:

Preparation: The mesenteric lymph nodes, after removal of surrounding adipose tissue, were placed in a 50 mL centrifuge tube filled with PBS (calcium-free) and penicillin–streptomycin. The tube was vigorously shaken to remove any surface pathogens from the lymph nodes.

Tissue Processing: The lymph nodes were finely minced and thoroughly ground.

Enzymatic Digestion: The chopped lymph node tissue was then incubated in PBS with collagenase IV at a 1:100 dilution at 37 °C for 30 min.

Termination of Digestion: To stop the digestion, serum was added to the mixture, which was then filtered through a 70 μm cell strainer and centrifuged at 800 rpm for 10 min.

### Effect of LF on DCs phenotype and cytokine secretion

DCs were treated with varying concentrations of LF. After 24 h, DCs were harvested for phenotype analysis. The study employed APC-conjugated MHC-II (MCA2314F, Bio-Rad, USA) along with PE-conjugated CD40 (12-0401-82, eBioscience, USA) and CD80 (12-0801-82, eBioscience) antibodies to evaluate the influence of LF on the phenotype of piglet DCs. Both cells and supernatants were collected for RT-qPCR and ELISA assays to assess changes in the expression and levels of IL-1β and IL-6, respectively.

### Mixed lymphocyte assay

Isolated T lymphocytes were labeled with carboxyfluorescein succinimidyl ester (CFSE, 565082, BD, USA) and incubated at 37 °C for 8 min before being washed twice with culture medium. The CFSE-labeled T cells were then mixed with DCs that had been treated with LF at ratios of 1:1 or 1:5. This mixture was incubated in a 37 °C incubator for 5 days. Flow cytometry was subsequently used to measure the CFSE fluorescence intensity in order to assess the effect of LF on the antigen presentation capability of the DCs.

### Surface plasmon resonance (SPR)

The SPR experiments were conducted at room temperature using a Biacore ATC-018 S200 system (GE Healthcare, USA). HSPG or PEDV S was covalently immobilized on a CM5 sensor chip. A HEPES buffer composed of 26.25 mM HEPES, 105 mM NaCl, 3.15 mM dithiothreitol (DTT), and 5.25 mM CaCl₂ was used for both the immobilization process and binding assays. One flow cell (Fc2) featured immobilized LF or HSPG, whereas the other (Fc1) served as a reference surface to correct for nonspecific interactions. The interaction between the immobilized LF or HSPG and the PEDV S protein was then investigated further.

### Plaque reduction neutralization assay

Vero E6 cells were seeded in 12-well cell culture plates and grown to approximately 90% confluency at 37 °C in a 5% CO_2_ humidified incubator for subsequent experiments. The supernatants from various treatment groups were first diluted tenfold with blank DMEM culture medium. The cell monolayers were then rinsed three times with blank DMEM to eliminate residual media, followed by the addition of the diluted supernatants. The plates were incubated at 37 °C in a 5% CO_2_ humidified environment for 1 h. After the incubation period, the supernatants were removed, and the cells were washed with blank DMEM to eliminate any remaining dilution fluid. A blend of 2 × DMEM and 1.6% sterile low-melting agarose was prepared at a 1:1 ratio, spread evenly over the cells, and allowed to solidify at room temperature. The plates were then returned to the incubator at 37 °C with 5% CO_2_ for cultivation. Once visible plaques formed (approximately 48–72 h later), the cells in each well were fixed with 4% paraformaldehyde at room temperature for one hour. The paraformaldehyde and agarose blocks were subsequently removed from the cell surface. The cells were then stained with 0.5% crystal violet for two hours, rinsed with running water to remove any excess stain, and left to air-dry. Finally, plaques were counted and photographed for analysis.

### Detection of PEDV viral load in piglet feces and tissues

Sterile cotton swabs were employed to collect fecal samples from the anal mucosa of piglets. These swabs were then centrifuged at 12 000 rpm for 15 min to separate the supernatant. Similarly, piglet tissues were processed by grinding and centrifugation to acquire the supernatant. Following this, 500 μL of Trizol reagent was added to each sample, and RNA was extracted using the standard Trizol method to isolate total cellular RNA. The RNA concentrations from various treatment groups were measured using a UV spectrophotometer (840-317400, Thermo Scientific™, USA), and the samples were diluted to maintain consistency across all groups. RNA normalization was then performed by reverse transcription using the HiScript TM QRT SuperMix kit according to the manufacturer's instructions to synthesize cDNA. Absolute quantification methods were used to quantify the PEDV viral load in the feces, employing a plasmid containing the PEDV-N gene sequence to construct a standard curve. The PEDV viral load in the piglet intestines was determined using relative quantitative PCR.

### Western blot analysis

The processing of tissue samples was carried out as described previously [12]: (1) A small amount of jejunal and ileal tissue was collected into a 2 mL centrifuge tube, to which 1 mL of PBS was added, and the tissue was homogenized using a tissue grinder for 1 min. The tube was then centrifuged to remove the PBS, followed by one more wash with 1 mL of PBS. After centrifugation at 12 000 r for 10 min, the PBS was discarded. This step was performed on ice. (2) Subsequently, 1 mL of pre-chilled RIPA lysis buffer was added, and the tissue was homogenized in the tissue grinder 3–5 times to ensure complete cell lysis. The centrifuge tube was placed on ice for 15 min, and the mixture was vortexed for 30 s every 5 min to facilitate complete lysis. (3) After lysis, the mixture was centrifuged at 12 000 r for 10 min at 4 °C, and the supernatant was transferred to a new 1.5 mL centrifuge tube for further analysis. Protein samples were then assessed through Western blotting using specific primary antibodies (PEDV-N, Medgenelabs Biological). Proteins were quantified using ImageJ software. Band intensity was measured and normalized against GAPDH expression.

### Data analysis

All experimental results are expressed as means ± standard deviation (means ± SD). The statistical evaluation of the data was performed using SPSS 17.0 software, utilizing one-way analysis of variance (One-Way ANOVA) to determine significance. Significance levels were denoted as follows: *P < 0.05, **P < 0.01.

## Results

### Effect of LF on PEDV replication

**Figure 1:**
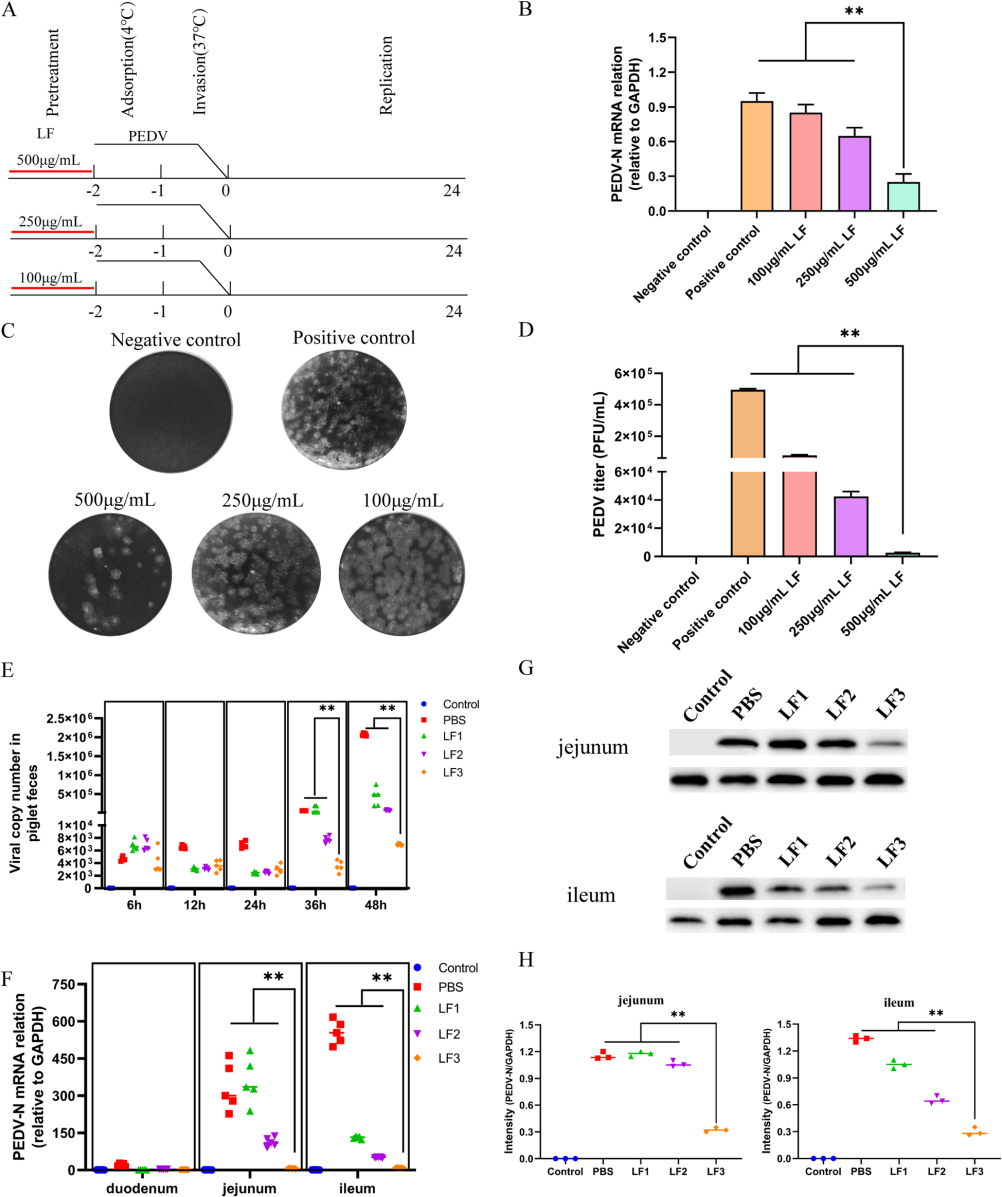
**LF inhibited PEDV replication**. **A** Schematic diagram depicting the antiviral effects of lactoferrin when pre-incubated with cells. **B**–**D** Quantification of viral RNA in cells (**B**) and viral titers in culture supernatants (**C** and **D**), determined using RT-qPCR and plaque reduction assays, respectively. **E** RT-qPCR analysis of viral RNA loads in feces from different piglet groups. **F**–**H** RT-qPCR and Western blot analyses showing viral RNA (**F**) and protein levels (**G** and **H**) in the jejunum and ileum of piglets from various groups. **P < 0.01.

The replication cycle of PEDV involves three stages: attachment, entry, and replication (Figure 1A). Prior investigations have shown that LF does not affect these stages directly. However, pre-incubation of LF with host cells before viral introduction markedly suppressed PEDV replication (Additional files 1A-C). Thus, we hypothesized that LF can modulate PEDV replication by interacting with the host cells. First, the influence of LF on host cell viability was measured via the CCK-8 assay, revealing that LF was non-toxic across various concentrations (Additional file 2A). Moreover, in vitro tests demonstrated that introducing LF during the virus attachment phase caused significant inhibition PEDV replication. This antiviral action of LF was found to be concentration-dependent, with higher doses enhancing the inhibition of viral replication (Figures 1B–D). In vivo experiments involved orally administering different doses of LF to piglets prior to PEDV infection. An acute toxicity assessment using a single oral gavage showed no mortality post oral administration LF (Additional file 3A), confirming the safety and lack of adverse effects of the administered LF concentrations. Clinical symptoms in PEDV-infected piglets were monitored. Those in the PBS and LF1 groups developed severe watery diarrhea within 48 h post-infection, and diarrheic piglets exhibited other clinical signs including lethargy, anorexia, and vomiting. In severe cases, yellow watery feces were observed contaminating the perianal region. Conversely, piglets receiving higher doses of LF (LF2 and LF3 groups) experienced notably milder symptoms. These piglets exhibited normal mental status and appetite, with only a few showing mild diarrhea characterized by well-formed feces. The severity of diarrhea was quantitatively evaluated through stool consistency scores, where higher scores indicated more severe diarrhea. Results indicated significantly lower diarrhea scores in the LF-treated groups compared to the PBS group, with the LF3 group showing the least severe symptoms (Additional file 3B). Furthermore, necropsy findings showed no pathological changes in the intestines of piglets in the uninfected control and LF3 groups, displaying normal intestinal contents and intact walls. However, those in the LF1 and PBS groups had notable intestinal abnormalities, including empty and thin, translucent walls (Additional file 3C). Finally, analysis of fecal viral load demonstrated a considerable reduction in viral shedding at 36 and 48 h post-infection among LF-treated groups (Figure 1E). RT-qPCR evaluation of viral distribution in the intestines showed that PEDV concentrations were significantly reduced in the jejunum and ileum of LF-treated piglets compared to the PBS-treated ones (Figure 1F). Additionally, Western blot analysis confirmed lower levels of PEDV proteins in these intestinal sections in LF-treated animals (Figures 1G, H).

### PEDV promoted viral replication by binding to HSPG on the cell surface

**Figure 2:**
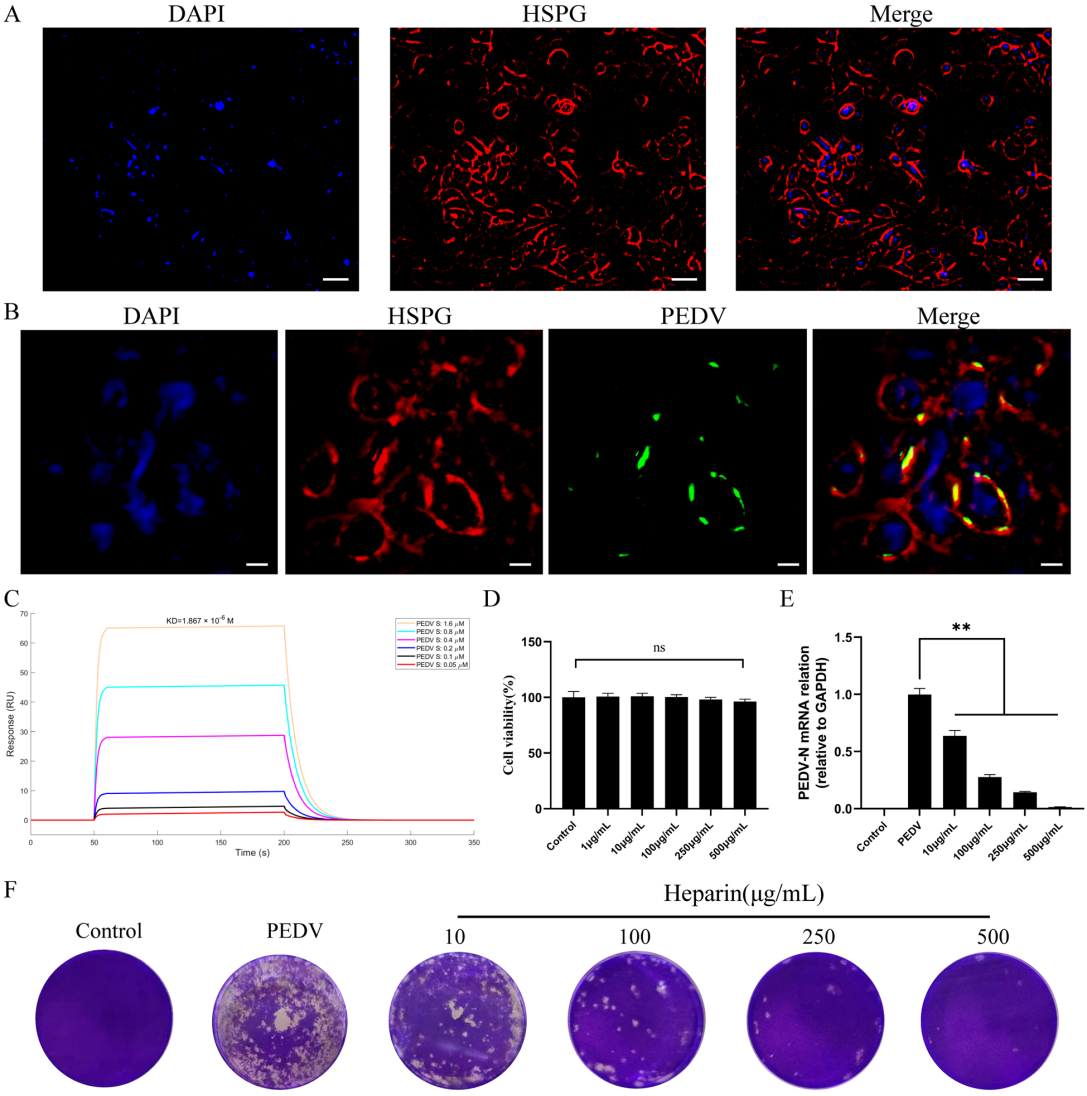
**PEDV promoted viral replication by binding to HSPG**. **A** Immunofluorescence analysis showing the distribution of HSPG in Vero E6 cells. HSPG appears red, while nuclei are counterstained blue with DAPI (scale bar = 100 μm). **B** Immunofluorescence analysis depicting the interaction between PEDV and HSPG, with HSPG stained red, PEDV-N protein stained green, and nuclei counterstained blue with DAPI (scale bar = 20 μm). **C** HSPG was covalently immobilized on a CM5 sensor chip, and the binding between HSPG and PEDV S was assessed by surface plasmon resonance assay. **D** Cytotoxicity assay evaluating the effect of various concentrations of HSPG on Vero E6 cells. **E**, **F** Measurement of viral RNA levels in cells (**E**) and viral titers in culture supernatants (**F**) conducted using RT-qPCR and plaque assays, respectively, for different experimental groups. ns, not significant, **P < 0.01.

HSPG is located on the cell surface, and the virus must first adsorb to it before invading the cell. Immunofluorescence analysis showed a significant distribution of HSPG on the surface of Vero cells (Figure 2A), and colocalization of HSPG with PEDV (Figure 2B). In SPR experiment, the results also indicated an interaction between PEDV S and HSPG (Figure 2C). To investigate the role of HSPG in PEDV replication, heparin, which acts as an HSPG inhibitor, was utilized. The initial step involved evaluating the cytotoxic effects of different heparin concentrations on cell viability via the CCK-8 assay, which indicated that higher doses of heparin were not cytotoxic (Figure 2D). Further experiments using RT-qPCR and plaque assays demonstrated that heparin effectively reduces PEDV replication in a dose-dependent manner (Figures 2E, F). These findings highlight the crucial role of HSPG in facilitating PEDV replication.

### LF inhibited PEDV replication by binding to HSPG

**Figure 3:**
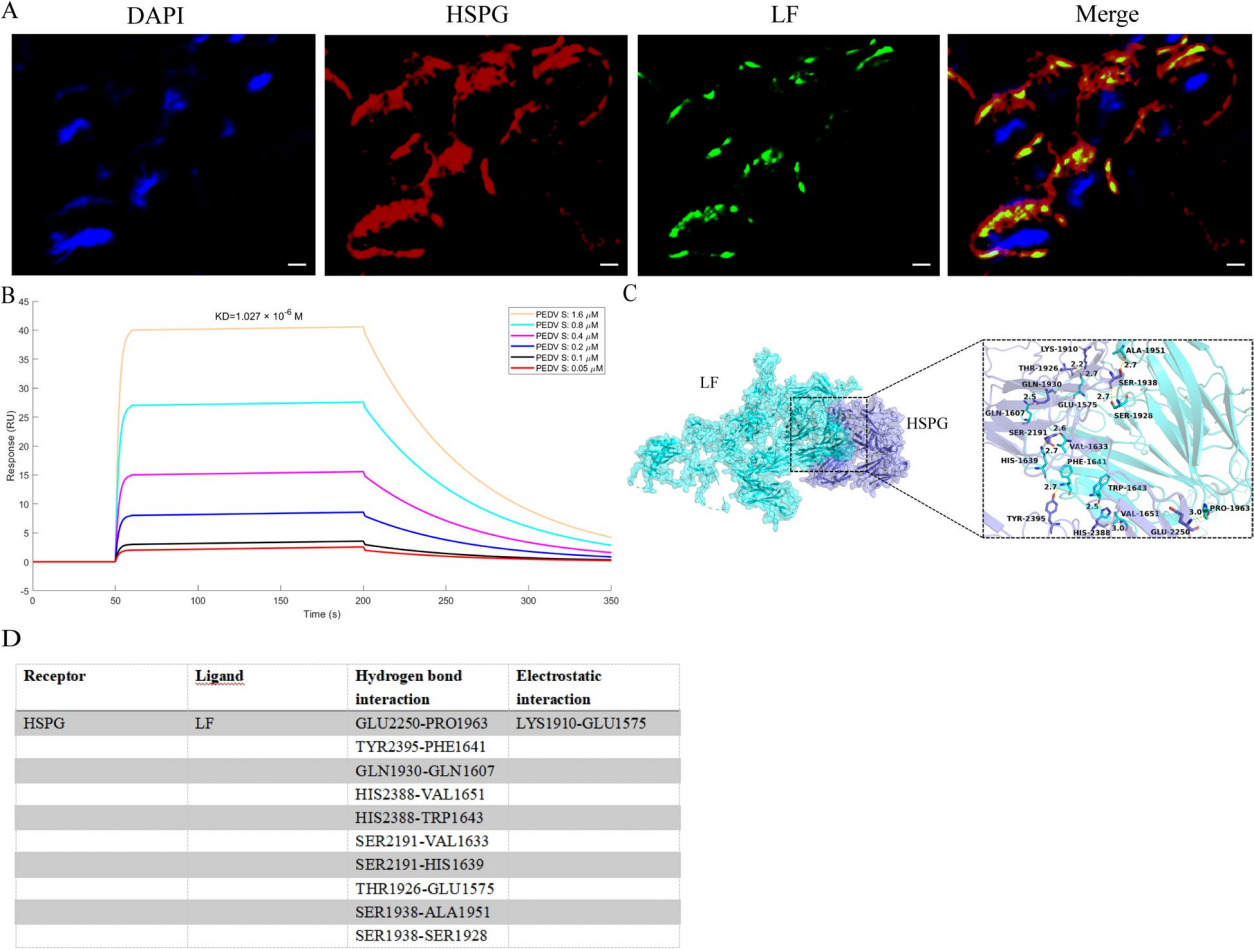
**There was an interaction between LF and HSPG**. **A** Immunofluorescence analysis displaying the distribution of HSPG in Vero E6 cells. HSPG is labeled in red using anti-HSPG antibodies, lactoferrin (LF) in green using anti-LF antibodies, and nuclei are stained with DAPI (blue) (scale bar = 20 μm). **B** HSPG was covalently immobilized on a CM5 sensor chip, and the binding between HSPG and LF was assessed by surface plasmon resonance assay. **C** Visualization of key binding sites between HSPG and LF, represented as stick structures in corresponding colors. **D** Identification and categorization of functional residues involved in HSPG-LF interactions, focusing on hydrogen bonding and electrostatic interactions, with interactions scored. The HSPG-LF binding sites are arranged in descending order based on their scores.

Immunofluorescence assays were utilized to examine the interaction between LF and HSPG, demonstrating their co-localization (Figure 3A). Furthermore, SPR kinetic analysis revealed a strong binding affinity between HSPG and LF, with an equilibrium dissociation constant (KD) of 1.027 × 10^ −6^M (Figure 3B). To further investigate this interaction, molecular docking was performed to predict their binding sites, it provided the optimal binding conformation between LF and HSPG (Figure 3C). Protein–protein interaction analysis revealed key functional residues involved in their binding (Figure 3D). Several residues facilitating hydrogen bonds were identified, including GLU2250, TYR2395, and GLN1930 on HSPG, along with PRO1963, PHE1641, and GLN1607 on LF. Electrostatic interactions were noted between LYS1910 on HSPG and GLU1575 on LF. The docking score, quantifying the binding strength, was determined to be -336.95. These results underscore the capability of LF to effectively bind HSPG.

**Figure 4:**
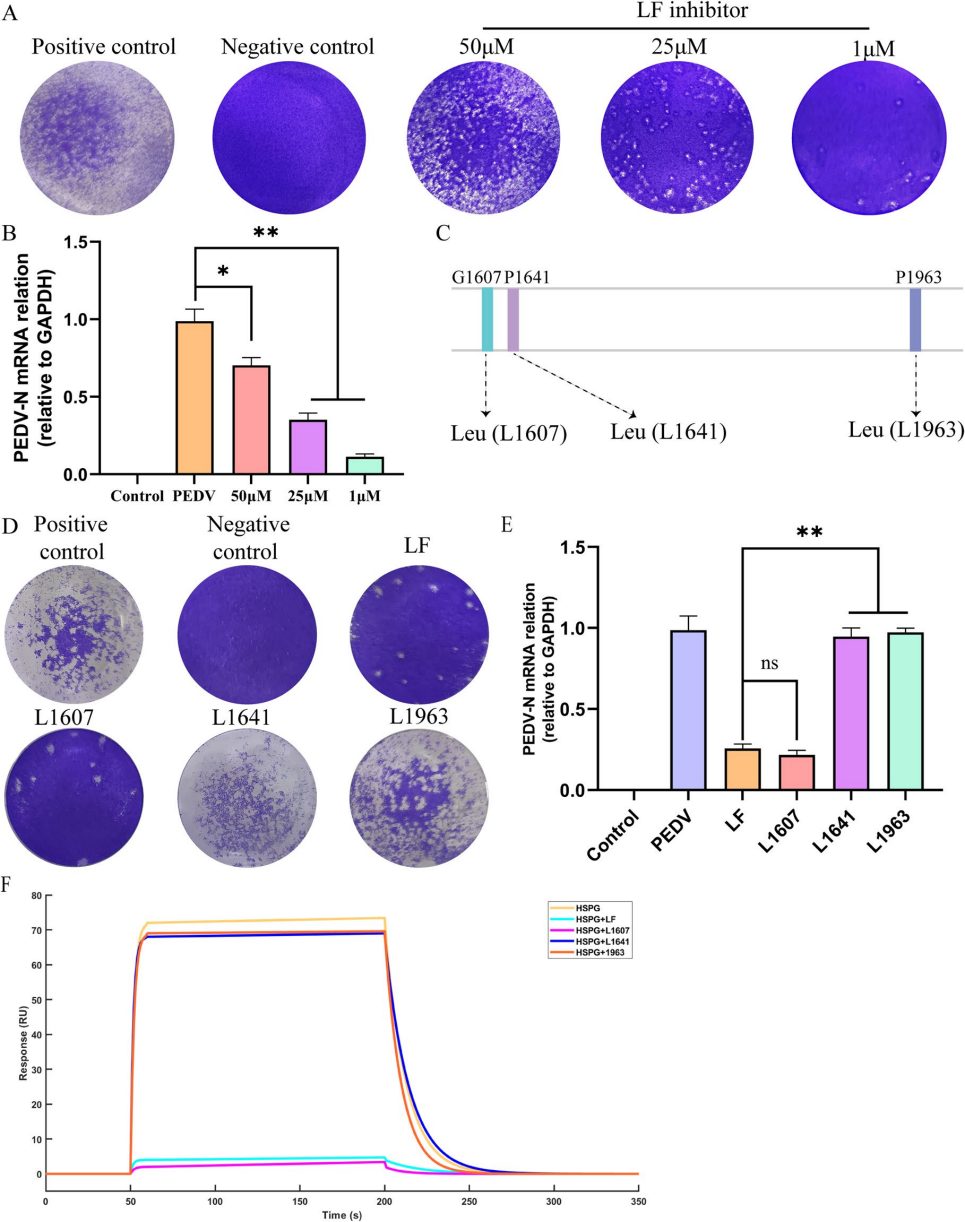
**LF competed with PEDV for binding to HSPG**. **A**, **B** Evaluation of the effects of varying concentrations of lactoferrin inhibitors on PEDV replication using plaque assays (**A**) and RT-qPCR (**B**). **C** Schematic illustration of LF mutants designed based on interaction sites. **D**–**F** Analysis of viral RNA levels in cells (**E**) and virus titers in supernatants (**D**) for different LF mutant treatment groups, assessed using plaque assays (**D**) and RT-qPCR (**E**). **F** Assessment of the interaction between HSPG and immobilized PEDV S protein on a CM5 sensor chip in the presence of LF or LF mutants, measured by surface plasmon resonance assay. ns, not significant, *P < 0.05, **P < 0.01.

We employed LF inhibitors and generated LF mutants to examine the effect of LF on PEDV replication through its interaction with HSPG. Initially, the cytotoxicity of these LF inhibitors was tested using a CCK-8 assay, which confirmed their non-toxic nature at various concentrations (Additional file 2B). Further analysis using RT-qPCR and plaque assays showed that introducing LF inhibitors into cells resulted in enhanced PEDV replication (Figures 4A and B). To further investigate how LF affects PEDV replication through HSPG interaction, LF mutants were created based on the molecular docking results (Figure 4C). These mutants were then tested to evaluate their influence on PEDV replication. The results indicated that mutants with specific point mutations at PRO1963 (L1963) and PHE1641 (L1641) failed to inhibit PEDV replication (Figures 4D and E), suggesting that modifications at these sites can disrupt the ability of LF to bind HSPG, thereby promoting PEDV replication. Furthermore, when LF was incubated with HSPG, it could not bind to the PEDV S protein. In contrast, the LF mutants (L1963 and L1641) maintained their capacity to bind the PEDV S protein even when incubated with HSPG (Figure 4F). This suggested that the L1963 and L1641 residues were key interaction sites for the binding between LF and HSPG, and that LF competitively inhibited PEDV from binding to HSPG on the cell surface.

### LF promoted maturation of porcine DCs

**Figure 5:**
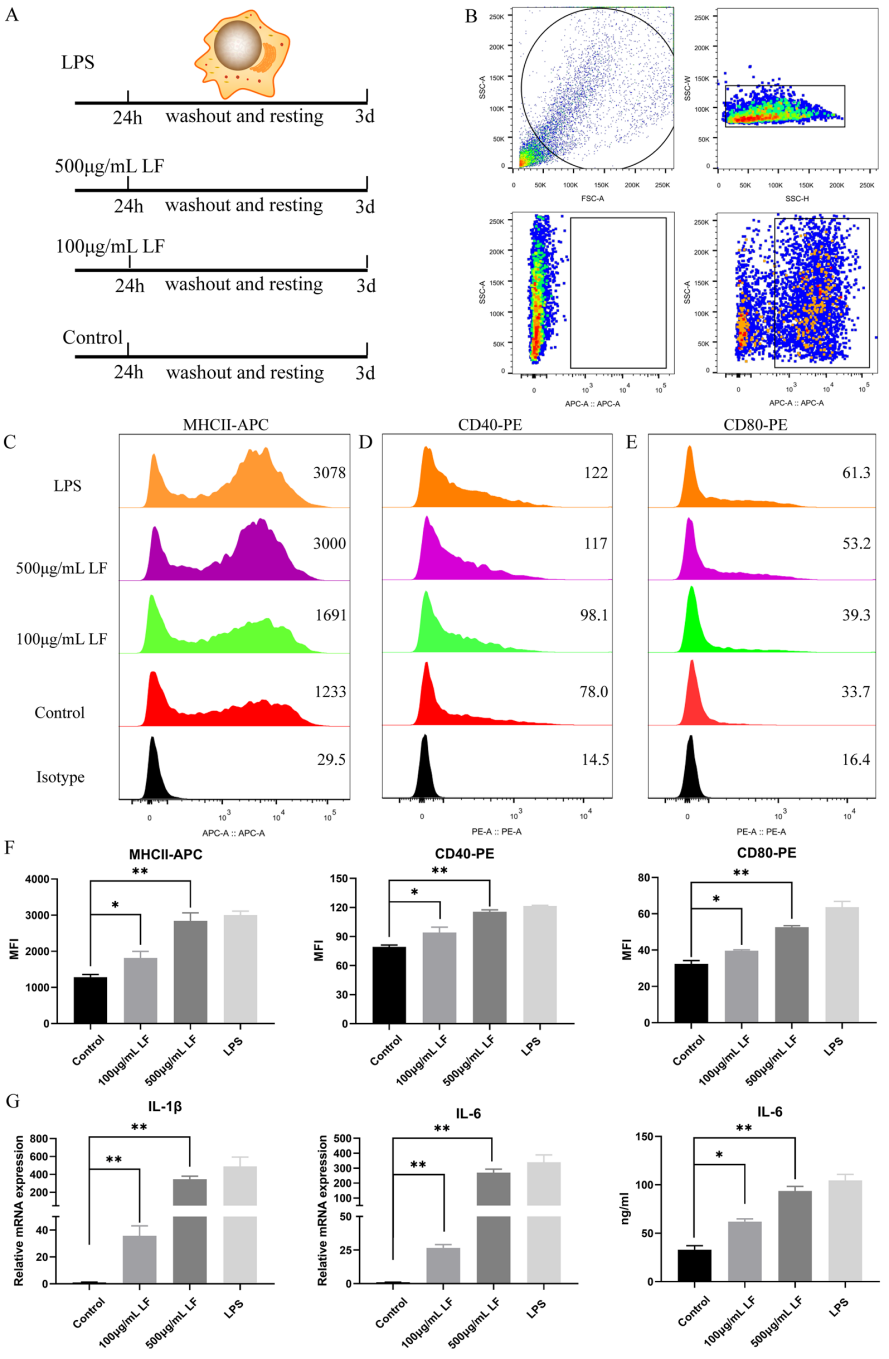
**LF affected the maturation of DCs**. **A** Schematic representation of DCs treated with varying concentrations of LF. **B**, **C** Flow cytometry analysis showing the expression levels of MHCII, CD40, and CD80 on the surfaces of DCs. **D** Evaluation of IL-1β and IL-6 mRNA levels in cells, along with the concentration of IL-6 in supernatants, measured using RT-qPCR and ELISA, respectively. *P < 0.05, **P < 0.01.

Upon maturation, DCs exhibit increased expression of MHCII and CD40, along with higher levels of the co-stimulatory molecule CD80. In this study, we used flow cytometry to evaluate the influence of LF on DC maturation (Figure 5A). The results indicated that LF significantly upregulated the surface expression of MHCII, CD40, and CD80 on DCs (Figures 5B–F), suggesting that LF can facilitate the phenotypic maturation of DCs. In addition to these phenotypic alterations, functionally mature DCs are known to produce various cytokines. We therefore measured cytokine production in DCs following different treatments using RT-qPCR and ELISA. As shown in Figure 5G, LF significantly enhanced the secretion of IL-1β and IL-6 by DCs, further supporting the role of LF in promoting the functional maturation of DCs.

### LF enhanced the antigen-presenting capacity of porcine DCs

**Figure 6:**
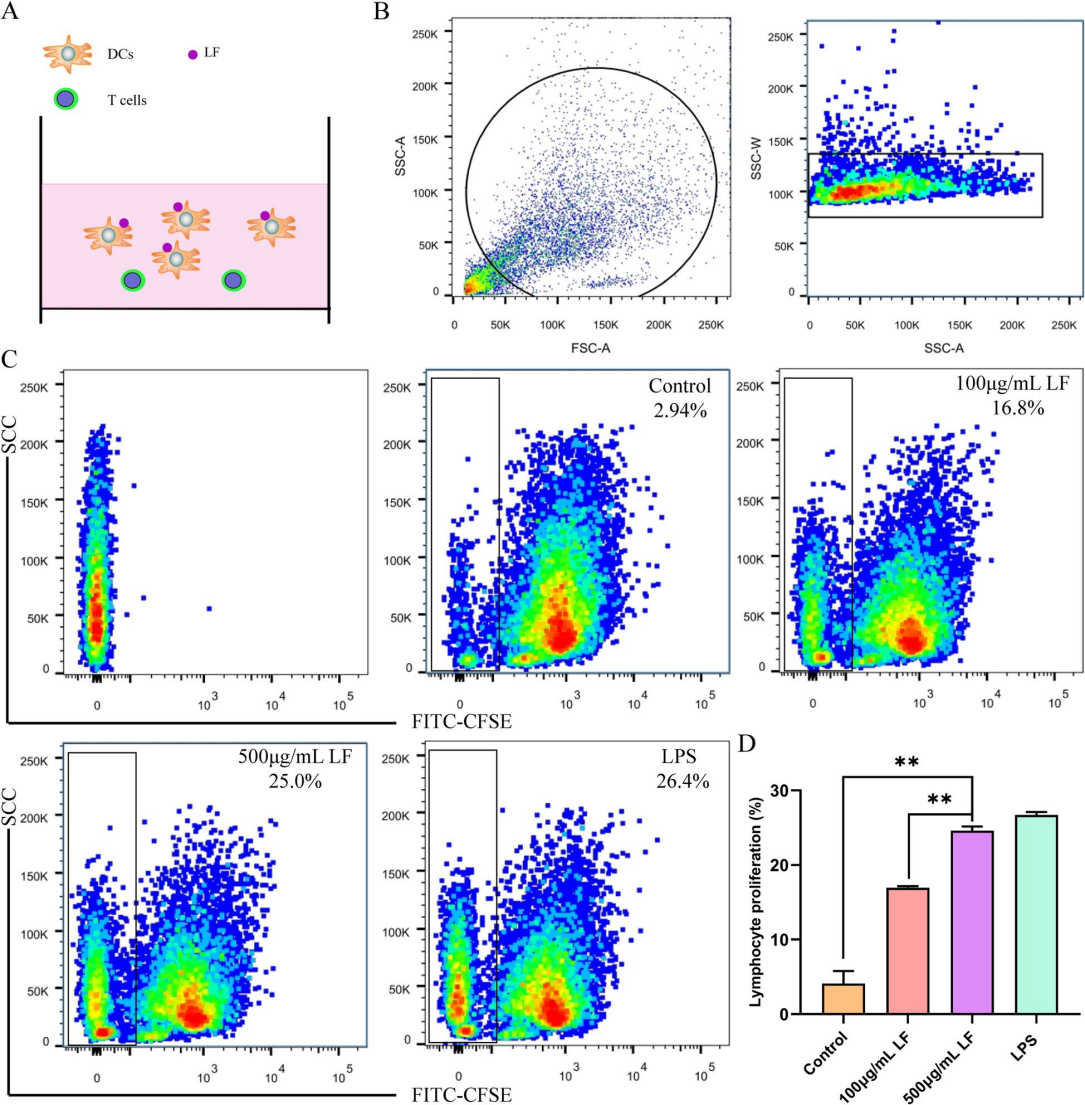
**LF boosted DCs antigen presentation**. **A** Schematic diagram depicting the effect of different concentrations of lactoferrin (LF) on T lymphocyte proliferation. **B**, **C** Flow cytometric analysis evaluating T lymphocyte proliferation across various LF concentrations. **D** Statistical analysis of mean fluorescence intensity, with significant differences between groups assessed using one-way ANOVA. **P < 0.01.

DCs are highly potent antigen-presenting cells that capture antigens at mucosal sites and then migrate to draining lymph nodes to activate naive T lymphocytes, facilitating their proliferation. In our study, we explored the impact of LF on the antigen-presenting capability of DCs through an in vitro mixed lymphocyte reaction assay. DCs were cultured and stimulated with varying concentrations of LF and LPS, then co-cultured with allogeneic lymphocytes (labeled with CFSE) at a 1:5 ratio for a mixed lymphocyte reaction (Figure 6A). The findings demonstrated that T lymphocytes co-cultured with LF-stimulated DCs showed significantly enhanced proliferation, with increased proliferation observed at higher LF concentrations (Figures 6B–D). This indicates that LF can enhance the ability of DCs to induce T lymphocyte proliferation.

## Discussion

Pig placentas are classified as epitheliochorial, which blocks the transfer of immunoglobulins and immune cells from the mother to the fetus [2]. Consequently, newborn piglets rely solely on passive immune protection obtained through the sow's milk, especially from colostrum [13]. Sow colostrum is rich in LF, a protein known for its antibacterial and immunomodulatory properties, its role in facilitating iron absorption in the intestines, and its contribution to neurological development among other functions [7, 14, 15]. Importantly, LF in colostrum also possesses significant antiviral activity [6, 15]. This study demonstrated through both in vivo and in vitro experiments that porcine LF can inhibit PEDV replication. Additionally, other studies have also indicated that bovine LF can block PEDV entry, albeit using LF from bovine milk [10]. In the context of swine farming, piglets typically receive LF through maternal colostrum, suggesting that boosting LF levels in colostrum might improve piglet resistance to PEDV infection.

LF primarily blocks virus entry by attaching to cell surface receptors or viral particles, thus preventing their interaction with host cells [16, 17]. Based on its role in Severe Acute Respiratory Syndrome Coronavirus 2 (SARS-CoV-2) replication [18–21], we hypothesized that its potential mechanisms in inhibiting PEDV replication include: (1) binding to the PEDV S protein, which may obstruct the virus's attachment to host cell surfaces; (2) attaching to cell surface HSPG, which are widely distributed and heavily sulfated on cell membranes, carrying significant negative charges, thus blocking interaction of PEDV with these cells. The glycosaminoglycan surface molecules of LF can electrostatically bind to the negatively charged HSPG, preventing virus-cell binding; (3) potentially enhancing the Type I interferon system to activate host antiviral responses. However, in this study, the co-incubation of LF and PEDV in Vero cells did not alter virus replication, indicating that LF does not inhibit PEDV replication by binding to the PEDV S protein. Additionally, since Vero cells do not produce interferon (IFN), indicating LF also does not inhibit PEDV replication through the IFN system. Therefore, LF likely inhibited PEDV replication primarily by competing with the virus for binding to cell surface HSPG receptors. This conclusion is supported by findings similar to those in Jonathan Z Sexton’s research [22], which demonstrated LF ability to inhibit SARS-CoV-2 replication by binding to HSPG.

LF not only has antiviral properties but also plays a vital role in immune modulation. Neonatal piglets receive LF through suckling colostrum, which is internalized into intestinal epithelial cells via LF receptors, primarily through megalin-mediated endocytosis [23]. Subsequently, LF is released into the lamina propria of the intestinal mucosa [23]. Beneath this layer, numerous DCs are widely distributed. DCs are pivotal antigen-presenting cells in host immune responses, continually surveilling for invading pathogens [24]. During viral infections of piglet intestines, DCs extend their dendrites through the gaps between epithelial cells to capture antigens from the lumen [24]. These antigens are processed and transported to mesenteric lymph nodes, where they are presented to T lymphocytes, making DCs a critical bridge between innate and adaptive mucosal immunity [25]. Our findings suggested that LF in sow colostrum induced DC maturation and boosted their ability to promote T lymphocyte proliferation, thereby potentially enhancing the intestinal mucosal immune response in neonatal piglets and contributing to their resistance against PEDV infection.

PEDV invasion of host cells involves two critical steps: virus attachment to host cells and subsequent binding to host cell receptors [26, 27]. Our study revealed that LF effectively impeded PEDV attachment to host cells. However, the impact of LF on PEDV binding to host cell receptors remains unclear. Potential receptors for PEDV entry include aminopeptidase N (APN), sialic acids, and tight junction proteins [28–30]. Although APN has been traditionally considered the primary entry receptor for PEDV, experiments that manipulated APN expression levels in MDCK cells did not significantly alter PEDV susceptibility, and PEDV spike proteins do not co-localize with APN [31, 32]. Therefore, there are ongoing uncertainties regarding APN's exclusive role as PEDV’s entry receptor. The current limitations in accurately identifying PEDV’s specific entry receptors constrain our ability to fully assess LF’s influence on PEDV entry receptors in this study.

## Supplementary Information

**Additional file 1. The role of LF in different stages of the viral replication cycle**.

**Additional file 2.** **The cytotoxic effects of LF and LF inhibitors in Vero E6 cells**.

**Additional file 3.** **Safety evaluation of orally LF in mice and the diarrhea scores and clinical symptoms of piglets in animal experiment**.

## Data Availability

The data that support the findings of this study are available from the corresponding author upon reasonable request.

## References

[1] Jung K, Saif LJ, Wang Q (2020) Porcine epidemic diarrhea virus (PEDV): an update on etiology, transmission, pathogenesis, and prevention and control. Virus Res 286:198045 32502552 10.1016/j.virusres.2020.198045 PMC7266596

[2] Langel SN, Paim FC, Lager KM, Vlasova AN, Saif LJ (2016) Lactogenic immunity and vaccines for porcine epidemic diarrhea virus (PEDV): historical and current concepts. Virus Res 226:93–107 27212686 10.1016/j.virusres.2016.05.016 PMC7111331

[3] Langel SN, Paim FC, Alhamo MA, Lager KM, Vlasova AN, Saif LJ (2019) Oral vitamin A supplementation of porcine epidemic diarrhea virus infected gilts enhances IgA and lactogenic immune protection of nursing piglets. Vet Res 50:101 31783923 10.1186/s13567-019-0719-y PMC6884901

[4] Gao Q, Zheng Z, Wang H, Yi S, Zhang G, Gong L (2021) The new porcine epidemic diarrhea virus outbreak may mean that existing commercial vaccines are not enough to fully protect against the epidemic strains. Front Vet Sci 8:697839 34291104 10.3389/fvets.2021.697839 PMC8287018

[5] Goede D, Murtaugh MP, Nerem J, Yeske P, Rossow K, Morrison R (2015) Previous infection of sows with a “mild” strain of porcine epidemic diarrhea virus confers protection against infection with a “severe” strain. Vet Microbiol 176:161–164 25601801 10.1016/j.vetmic.2014.12.019

[6] Florisa R, Recio I, Berkhout B, Visser S (2003) Antibacterial and antiviral effects of milk proteins and derivatives thereof. Curr Pharm Des 9:1257–1275 12769735 10.2174/1381612033454810

[7] Salmon H, Berri M, Gerdts V, Meurens F (2009) Humoral and cellular factors of maternal immunity in swine. Dev Comp Immunol 33:384–393 18761034 10.1016/j.dci.2008.07.007

[8] Segura M, Martínez-Miró S, López MJ, Madrid J, Hernández F (2020) Effect of parity on reproductive performance and composition of sow colostrum during first 24 h postpartum. Animals 10:1853 33053679 10.3390/ani10101853 PMC7601285

[9] Jahan M, Francis N, Wang B (2020) Milk lactoferrin concentration of primiparous and multiparous sows during lactation. J Dairy Sci 103:7521–7530 32448579 10.3168/jds.2020-18148

[10] Zhou H, Li X, Wang Z, Yin J, Tan H, Wang L, Qiao X, Jiang Y, Cui W, Liu M, Li Y, Xu Y, Tang L (2018) Construction and characterization of thymidine auxotrophic (ΔthyA) recombinant *Lactobacillus casei* expressing bovine lactoferricin. BMC Vet Res 14:206 29945678 10.1186/s12917-018-1516-y PMC6020375

[11] Han X, Gao Y, Li G, Xiong Y, Zhao C, Ruan J, Ma Y, Li X, Li C, Zhao S, Xie S (2020) Enhancing the antibacterial activities of sow milk via site-specific knock-in of a lactoferrin gene in pigs using CRISPR/Cas9 technology. Cell Biosci 10:133 33292594 10.1186/s13578-020-00496-y PMC7678085

[12] Li Y, Wang X, Zhang E, Liu R, Yang C, Duan Y, Jiang Y, Yang Q (2022) Calpain-1: a novel antiviral host factor identified in porcine small intestinal mucus. MBio 13:e0035822 36102516 10.1128/mbio.00358-22 PMC9600339

[13] Amimo JO, Michael H, Chepngeno J, Jung K, Raev SA, Paim FC, Lee MV, Damtie D, Vlasova AN, Saif LJ (2024) Maternal immunization and vitamin A sufficiency impact sow primary adaptive immunity and passive protection to nursing piglets against porcine epidemic diarrhea virus infection. Front Immunol 15:1397118 38812505 10.3389/fimmu.2024.1397118 PMC11133611

[14] Abd El-Hack ME, Abdelnour SA, Kamal M, Khafaga AF, Shakoori AM, Bagadood RM, Naffadi HM, Alyahyawi AY, Khojah H, Alghamdi S, Jaremko M, Świątkiewicz S (2023) Lactoferrin: antimicrobial impacts, genomic guardian, therapeutic uses and clinical significance for humans and animals. Biomed Pharmacother 164:114967 37290189 10.1016/j.biopha.2023.114967

[15] Andreu S, Ripa I, Bello-Morales R, López-Guerrero JA (2023) Liposomal lactoferrin exerts antiviral activity against HCoV-229E and SARS-CoV-2 pseudoviruses in vitro. Viruses 15:972 37112952 10.3390/v15040972 PMC10142420

[16] Kell DB, Heyden EL, Pretorius E (2020) The biology of lactoferrin, an iron-binding protein that can help defend against viruses and bacteria. Front Immunol 11:1221 32574271 10.3389/fimmu.2020.01221 PMC7271924

[17] Campione E, Lanna C, Cosio T, Rosa L, Conte MP, Iacovelli F, Romeo A, Falconi M, Del Vecchio C, Franchin E, Lia MS, Minieri M, Chiaramonte C, Ciotti M, Nuccetelli M, Terrinoni A, Iannuzzi I, Coppeda L, Magrini A, Bernardini S, Sabatini S, Rosapepe F, Bartoletti PL, Moricca N, Di Lorenzo A, Andreoni M, Sarmati L, Miani A, Piscitelli P, Valenti P, Bianchi L (2021) Lactoferrin against SARS-CoV-2: in vitro and in silico evidences. Front Pharmacol 12:666600 34220505 10.3389/fphar.2021.666600 PMC8242182

[18] Lang J, Yang N, Deng J, Liu K, Yang P, Zhang G, Jiang C (2011) Inhibition of SARS pseudovirus cell entry by lactoferrin binding to heparan sulfate proteoglycans. PLoS One 6:e23710 21887302 10.1371/journal.pone.0023710 PMC3161750

[19] Takakura N, Wakabayashi H, Yamauchi K, Takase M (2006) Influences of orally administered lactoferrin on IFN-gamma and IL-10 production by intestinal intraepithelial lymphocytes and mesenteric lymph-node cells. Biochem Cell Biol 84:363–368 16936808 10.1139/o06-056

[20] Campione E, Cosio T, Rosa L, Lanna C, Di Girolamo S, Gaziano R, Valenti P, Bianchi L (2020) Lactoferrin as protective natural barrier of respiratory and intestinal mucosa against coronavirus infection and inflammation. Int J Mol Sci 21:4903 32664543 10.3390/ijms21144903 PMC7402319

[21] Sarrazin S, Lamanna WC, Esko JD (2011) Heparan sulfate proteoglycans. Cold Spring Harb Perspect Biol 3:a004952 21690215 10.1101/cshperspect.a004952 PMC3119907

[22] Mirabelli C, Wotring JW, Zhang CJ, McCarty SM, Fursmidt R, Pretto CD, Qiao Y, Zhang Y, Frum T, Kadambi NS, Amin AT, O’Meara TR, Spence JR, Huang J, Alysandratos KD, Kotton DN, Handelman SK, Wobus CE, Weatherwax KJ, Mashour GA, O’Meara MJ, Chinnaiyan AM, Sexton JZ (2021) Morphological cell profiling of SARS-CoV-2 infection identifies drug repurposing candidates for COVID-19. Proc Natl Acad Sci USA 118:e2105815118 34413211 10.1073/pnas.2105815118 PMC8433531

[23] Matsuzaki T, Nakamura M, Nogita T, Sato A (2019) Cellular uptake and release of intact lactoferrin and its derivatives in an intestinal enterocyte model of caco-2 cells. Biol Pharm Bull 42:989–995 31155596 10.1248/bpb.b19-00011

[24] Ueno H, Klechevsky E, Schmitt N, Ni L, Flamar AL, Zurawski S, Zurawski G, Palucka K, Banchereau J, Oh S (2011) Targeting human dendritic cell subsets for improved vaccines. Sem Immunol 23:21–27 10.1016/j.smim.2011.01.004 PMC3071344 21277223

[25] Spadaro M, Caorsi C, Ceruti P, Varadhachary A, Forni G, Pericle F, Giovarelli M (2008) Lactoferrin, a major defense protein of innate immunity, is a novel maturation factor for human dendritic cells. FASEB J 22:2747–2757 18364398 10.1096/fj.07-098038

[26] Wicht O, Li W, Willems L, Meuleman TJ, Wubbolts RW, van Kuppeveld FJ, Rottier PJ, Bosch BJ (2014) Proteolytic activation of the porcine epidemic diarrhea coronavirus spike fusion protein by trypsin in cell culture. J Virol 88:7952–7961 24807723 10.1128/JVI.00297-14 PMC4097775

[27] Liu C, Ma Y, Yang Y, Zheng Y, Shang J, Zhou Y, Jiang S, Du L, Li J, Li F (2016) Cell entry of porcine epidemic diarrhea coronavirus is activated by lysosomal proteases. J Biol Chem 291:24779–24786 27729455 10.1074/jbc.M116.740746 PMC5114425

[28] Li BX, Ge JW, Li YJ (2007) Porcine aminopeptidase N is a functional receptor for the PEDV coronavirus. Virology 365:166–172 17467767 10.1016/j.virol.2007.03.031 PMC7103304

[29] Zhao S, Gao J, Zhu L, Yang Q (2014) Transmissible gastroenteritis virus and porcine epidemic diarrhoea virus infection induces dramatic changes in the tight junctions and microfilaments of polarized IPEC-J2 cells. Virus Res 192:34–45 25173696 10.1016/j.virusres.2014.08.014 PMC7114495

[30] Luo X, Guo L, Zhang J, Xu Y, Gu W, Feng L, Wang Y (2017) Tight junction protein occludin is a porcine epidemic diarrhea virus entry factor. J Virol 91:e00202-17 28275187 10.1128/JVI.00202-17 PMC5411586

[31] Li W, Luo R, He Q, van Kuppeveld FJM, Rottier PJM, Bosch BJ (2017) Aminopeptidase N is not required for porcine epidemic diarrhea virus cell entry. Virus Res 235:6–13 28363778 10.1016/j.virusres.2017.03.018 PMC7114539

[32] Ji CM, Wang B, Zhou J, Huang YW (2018) Aminopeptidase-N-independent entry of porcine epidemic diarrhea virus into Vero or porcine small intestine epithelial cells. Virology 517:16–23 29502803 10.1016/j.virol.2018.02.019 PMC7127557

