# Supplementary figures and images for: Lactoferrin exhibits PEDV antiviral activity by interfering with spike-heparan sulfate proteoglycans binding and activating mucosal immune response
Source: Vet Res. 2025 Jan 31;56:25. doi: 10.1186/s13567-025-01456-5 (PMC11786531; doi:10.1186/s13567-025-01456-5)

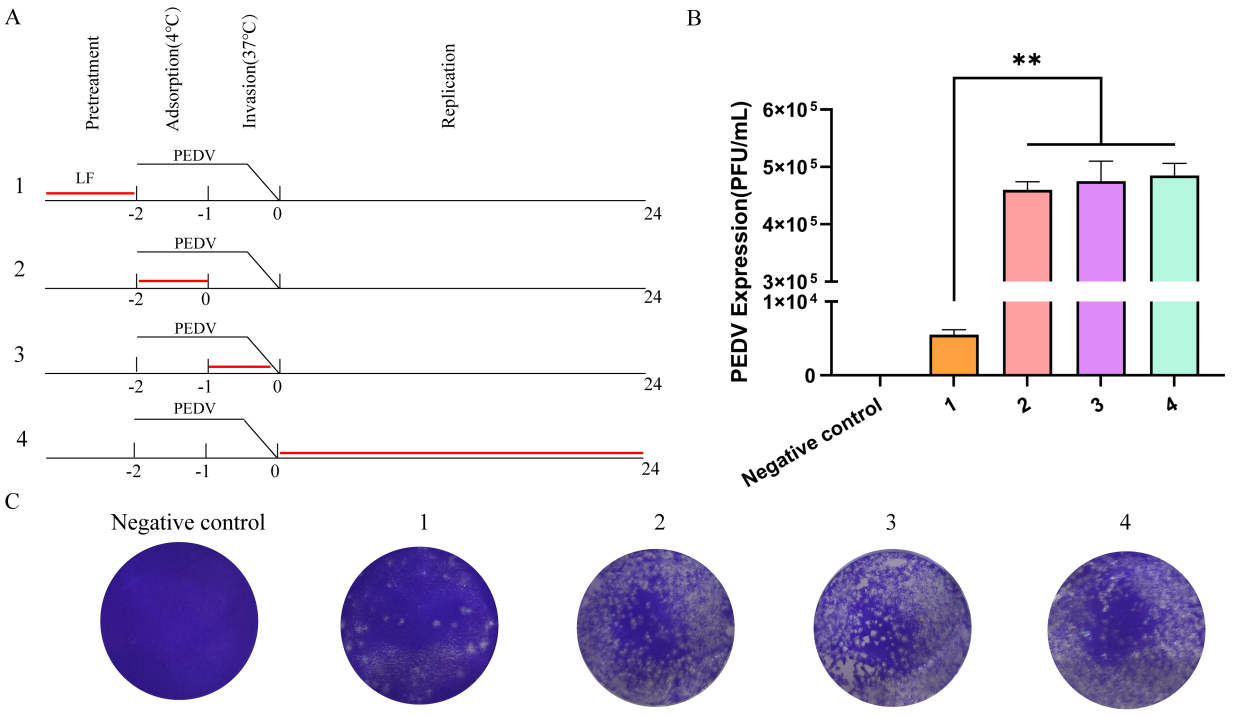

Supplement: Supplementary file 1 — Additional file 1. The role of LF in different stages of the viral replication cycle. [file 13567_2025_1456_MOESM1_ESM.docx]

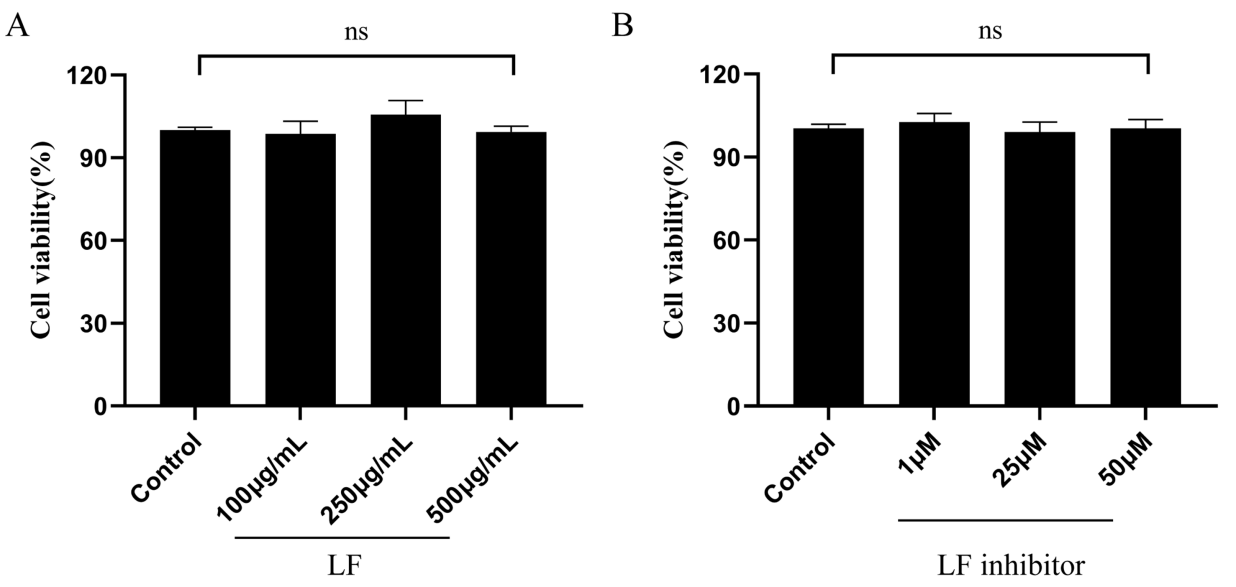

Supplement: Supplementary file 2 — Additional file 2. The cytotoxic effects of LF and LF inhibitors in Vero E6 cells. [file 13567_2025_1456_MOESM2_ESM.docx]

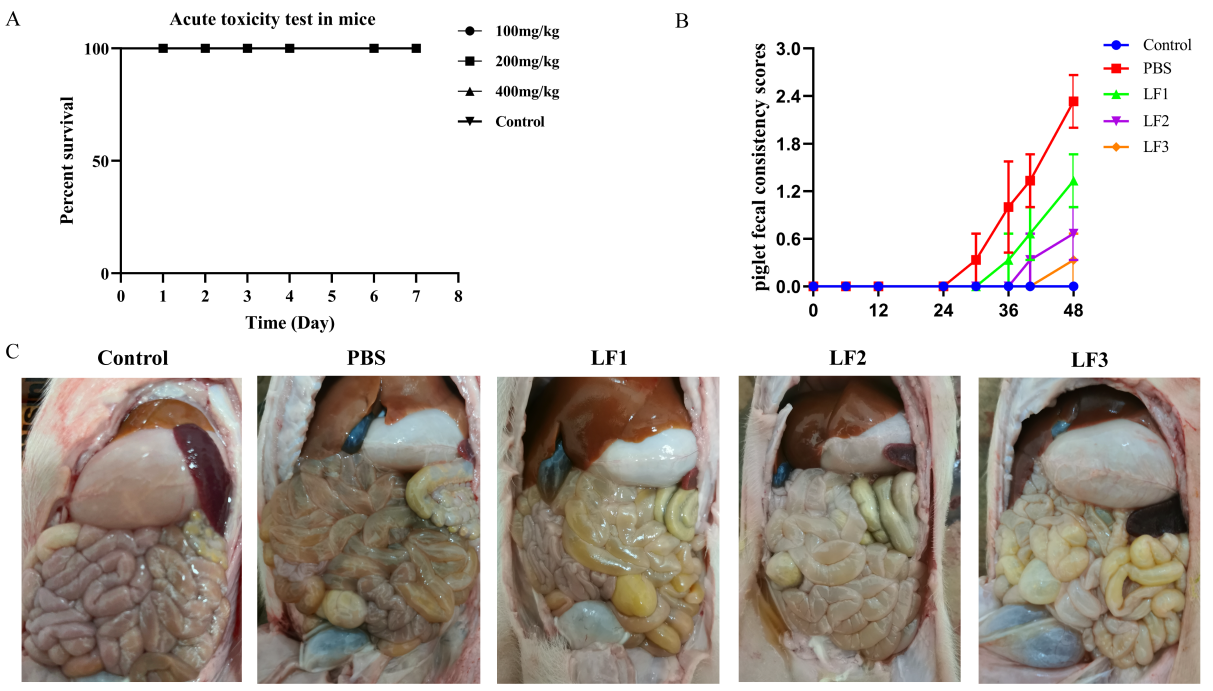

Supplement: Supplementary file 3 — Additional file 3. Safety evaluation of orally LF in mice and the diarrhea scores and clinical symptoms of piglets in animal experiment. [file 13567_2025_1456_MOESM3_ESM.docx]
